# Gallstone Ileus: A Rare Cause of Mechanical Bowel Obstruction

**DOI:** 10.7759/cureus.35588

**Published:** 2023-02-28

**Authors:** Sandra D Santos, João Louro, Carlos M Costa Almeida, Sandra Simões, Jorge Fortuna

**Affiliations:** 1 Internal Medicine, University Hospital Center of Coimbra, Coimbra, PRT; 2 General Surgery, Serviço de Cirurgia Geral, Hospital Dr. Nélio Mendonça, Funchal, Madeira, Portugal, Funchal, PRT; 3 General Surgery, Hospital Geral - Covões, University Hospital Center of Coimbra, Coimbra, PRT

**Keywords:** physical rehabilitation, elderly population, enterolithotomy, bowel obstruction, gallstone ileus

## Abstract

A gallstone ileus is a rare cause of mechanical bowel obstruction, accounting for 1% to 4% of all cases. Twenty-five percent of the patients are 65 years of age or older and often present previous significant medical conditions.

The authors report the case of an 87-year-old male patient, admitted with the diagnosis of community-acquired pneumonia, who later developed frequent episodes of biliary vomiting, intermittent constipation, and abdominal distension. Abdominal imaging (ultrasound and computed tomography (CT)) showed evidence of a localized inflammatory process in a small bowel loop but excluded vesicular lithiasis. After a failure in the medical approach with antibiotics, an exploratory laparotomy was performed, identifying the intestinal occlusion area, followed by an enterolithotomy at the specific area, and extraction of a 4 cm stone of acellular material. Posteriorly, the patient was treated for three weeks with a carbapenem and physical rehabilitation was promptly initiated with full recovery of his previous status.

Gallstone ileus is a very challenging diagnosis and surgery is the treatment of choice. In elderly patients, it is important to promote prompt physical rehabilitation to prevent prolonged bed rest.

## Introduction

A gallstone ileus is usually a late complication of gallbladder disease, being responsible for 1% to 4% of all mechanical intestinal obstructions, especially in elderly individuals [1,2]. It usually occurs in women and in patients with a history of choledocholithiasis [2]. It is caused by intestinal impaction of a gallstone that enters the bowel via a cholecystoenteric fistula. The mortality rate is considerable, ranging between 12% and 27%. Treatment in most cases is surgical, with a focus on resolving the intestinal obstruction. Clinically, it presents with the usual signs and symptoms of intestinal obstruction, and abdominal CT is the best diagnostic method [3]. The most effective treatment is the mechanical removal of the cause of the obstruction [2], usually by a surgical intervention [2], involving an enterolithotomy.

The authors present this case, not only for its rarity but also to highlight the clinical challenge that this condition implies and the importance of removing the bowel obstruction, as soon as possible, in the process of full recovery of the patient.

## Case presentation

An 87-year-old male, with a previous history of hypertension, diabetes, and benign prostate hyperplasia, not medicated due to therapeutic non-compliance, was admitted to the internal medicine ward with the diagnosis of community-acquired pneumonia, with global respiratory failure, which was treated with amoxicillin-clavulanic acid and azithromycin.

**Figure 1:**
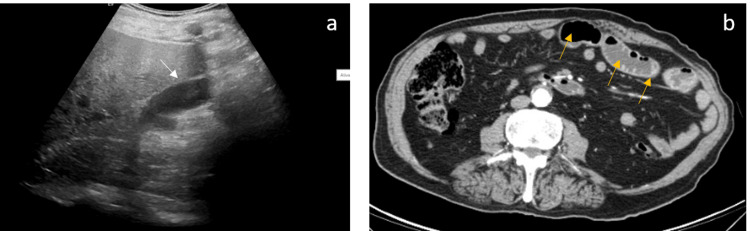
a) Abdominal ultrasound showing the gallbladder without lithiasis (white arrow) or fistulous tract; b) CT scan of the abdomen showing dilatation of the first jejunal loops with liquid and gas components and parietal thickening (yellow arrows)

On the second day of admission, he started biliary vomiting (six episodes/day), associated with exuberant abdominal distension, especially at the epigastric level, and intermittent constipation. A nasogastric tube was placed, draining a large volume of biliary content. He underwent abdominal radiography, which excluded air-fluid levels and the abdominal ultrasound (Figure 1a) showed thickening of a small bowel loop, with normal caliber, suggestive of inflammatory changes, excluding vesicular lithiasis. The patient was then treated with piperacillin-tazobactam (4500mg, q8h) and metronidazole (500mg, q8h). Three days later, due to the lack of significant clinical improvement, an abdominal CT scan was performed and showed dilatation of the first jejunal loops with liquid and gas components and parietal thickening (Figure 1b).

**Figure 2:**
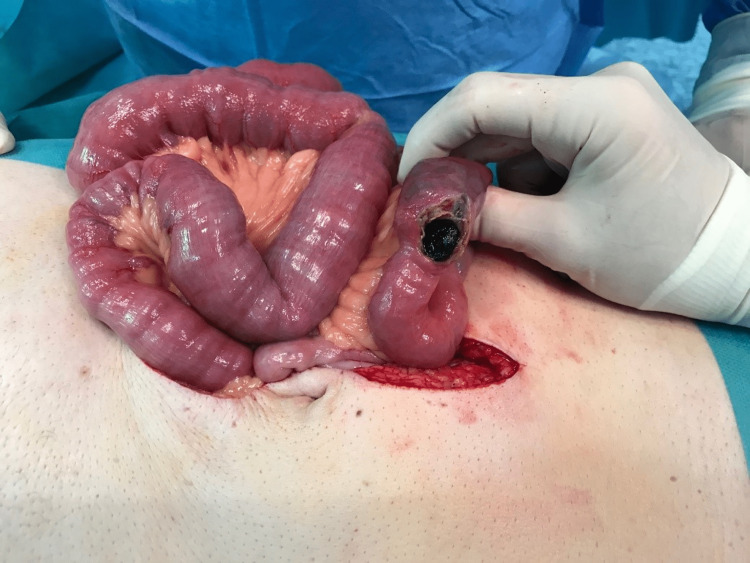
Gallstone extraction through enterolithotomy

**Figure 3:**
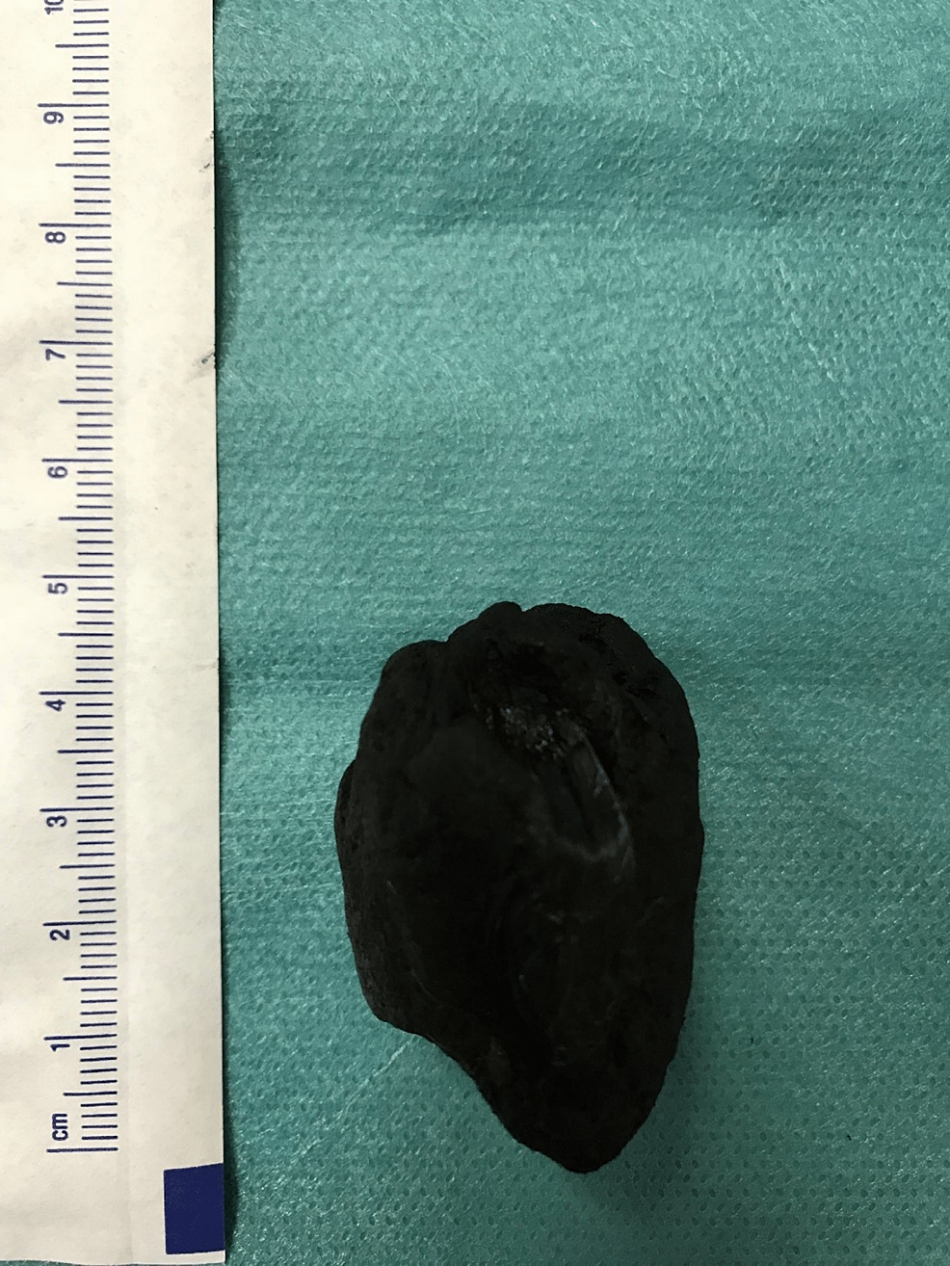
Gallstone measuring 4x3x2.5 cm extracted after enterolithotomy

Seven days after the onset of the symptoms, the patient was submitted to an exploratory laparotomy. The presence of distended intestinal loops with signs of venous stasis was observed to be adjacent to an intestinal occlusion area. At this specific region, an enterolithotomy was performed (Figure 2) with subsequent extraction of a 4 cm stone (Figure 3). Intraoperatively, nothing suspicious was found and, in this case, there is no indication to explore the gallbladder to find the fistulous tract and perform cholecystectomy due to the increased risk of postoperative fistula [4].

After surgery, the patient was transferred to the intensive care unit and started on meropenem (1g, q8h). After three days, he was extubated and transferred again to the internal medicine ward, where he completed 21 days of antibiotics, with gradual and sustained clinical improvement. The study of the excised stone identified acellular material. After one week, the patient showed a reduction in the inflammatory parameters, resumption of gastrointestinal transit, and started a normal diet, being able to begin the rehabilitation program. Posteriorly, he was admitted to a rehabilitation unit where he underwent intensive physical rehabilitation and was able to fully recover.

## Discussion

A gallstone ileus is a rare condition first described by Bartholin in 1645 during an autopsy [5]. It consists of a mechanical intestinal obstruction due to the impaction of one or more large gallstones, usually bigger than 2.5 cm, within the gastrointestinal tract [6,7]. The classical clinical presentation of gallstone ileus occurs typically in older women, with episodic subacute obstruction. Gallstone impaction usually occurs in an intermittent manner, producing diffuse abdominal pain and vomiting when impacting, alternating with a relief period when the gallstone becomes disimpacted, recurring again as the stone lodges in the more distal bowel lumen. As a result, vague and intermittent symptoms may be present for one to eight days prior to evaluation [8].

The main goal of treatment is the prompt relief of intestinal obstruction by removing the offending gallstone, with surgical intervention remaining the treatment of choice. Surgical treatment is still subject to debate and research. The current surgical techniques available are (1) enterolithotomy; (2) enterolithotomy with cholecystectomy performed later (two-stage surgery); (3) enterolithotomy, cholecystectomy, and fistula repair (one-stage surgery) [9].

Several factors should be considered in the choice of the most appropriate surgical approach. Preoperative conditions, such as the age of the patients, comorbidity, diagnostic delay, and need for urgent surgery have a significant impact on choosing the best approach, which should be decided by a careful evaluation of the risk-to-benefit ratio [9].

Reisner and Cohen compared mortality in patients who underwent enterotomy alone and patients who underwent one-stage surgery and found a mortality rate of 11.7% for patients who underwent enterotomy alone versus 16.9% for patients who had one-stage surgery [10,11]. Enterolithotomy via laparotomy is the most reported surgical procedure [7] and, at present, it is considered a good approach for patients with significant comorbidities, hemodynamic instability, or high-risk surgical dissection [11]. In the presented case, considering the age of the patient and his comorbidities, this was also the technique of choice.

Overall, the prognosis of gallstone ileus is poor, with mortality rates up to 20%, mainly because of the delayed diagnosis and coexistence of comorbid conditions, more frequent in the elderly population [12].

## Conclusions

The authors present a rare case of gallstone ileus in a male patient, in the absence of vesicular lithiasis or evidence of enteric fistula, which made the diagnosis very difficult. Despite the importance of clinical and imagiological findings, the crucial step for the patient's diagnosis and treatment was the surgical procedure. They also point out the importance of early rehabilitation, especially in the elderly population, which is fundamental to the full recovery and preservation of the functional status of these patients.

## References

[1] Pratas N, Salvador D, Costa CS (2020). Gallstone ileus caused by a gallstone impacted at a cecum neoplasm - a case report. Int J Surg Case Rep.

[2] Jakubauskas M, Luksaite R, Sileikis A, Strupas K, Poskus T (2019). Gallstone ileus: management and clinical outcomes. Medicina (Kaunas).

[3] Hussain J, Alrashed AM, Alkhadher T, Wood S, Behbehani AD, Termos S (2018). Gall stone ileus: Unfamiliar cause of bowel obstruction. Case report and literature review. Int J Surg Case Rep.

[4] Cameron JL, Cameron AM (2023). Current Surgical Therapy, 14th Edition. https://www.us.elsevierhealth.com/current-surgical-therapy-9780323796835.html.

[5] Masannat Y, Masannat Y, Shatnawei A (2006). Gallstone ileus: a review. Mt Sinai J Med.

[6] Deitz DM, Standage BA, Pinson CW, McConnell DB, Krippaehne WW (1986). Improving the outcome in gallstone ileus. Am J Surg.

[7] Abou-Saif A, Al-Kawas FH (2002). Complications of gallstone disease: Mirizzi syndrome, cholecystocholedochal fistula, and gallstone ileus. Am J Gastroenterol.

[8] Nuño-Guzmán CM, Marín-Contreras ME, Figueroa-Sánchez M, Corona JL (2016). Gallstone ileus, clinical presentation, diagnostic and treatment approach. World J Gastrointest Surg.

[9] Conzo G, Mauriello C, Gambardella C, Napolitano S, Cavallo F, Tartaglia E, Santini L (2013). Gallstone ileus: one-stage surgery in an elderly patient: one-stage surgery in gallstone ileus. Int J Surg Case Rep.

[10] Reisner RM, Cohen JR (1994). Gallstone ileus: a review of 1,001 reported cases. Am Surg.

[11] Vera-Mansilla C, Sanchez-Gollarte A, Matias B, Mendoza-Moreno F, Díez-Alonso M, Garcia-Moreno Nisa F (2022). Surgical treatment of gallstone ileus: less is more. Visc Med.

[12] Turner AR, Sharma B, Mukherjee S (2022). Gallstone Ileus. https://www.ncbi.nlm.nih.gov/books/NBK430834/.

